# P-738. A Microbiological Profile of Necrotizing Fasciitis at a Tertiary Referral Hospital in a Low/Middle Income Country

**DOI:** 10.1093/ofid/ofae631.934

**Published:** 2025-01-29

**Authors:** Caren Challita, Tamara Abdallah, Christopher Abi Zeid Daou, Tania Sawaya, Ali Hallal, Naji Madi, Nesrine Rizk

**Affiliations:** American University of Beirut Medical Center, Beirut, Beyrouth, Lebanon; American University of Beirut Medical Center, Beirut, Beyrouth, Lebanon; American University of Beirut, Beirut, Beyrouth, Lebanon; American University of Beirut, Beirut, Beyrouth, Lebanon; American University of Beirut, Beirut, Beyrouth, Lebanon; American University of Beirut, Beirut, Beyrouth, Lebanon; American University of Beirut, Beirut, Beyrouth, Lebanon

## Abstract

**Background:**

: Necrotizing fasciitis (NF), an uncommon, fatal infection of the skin and soft tissues, carries a significant mortality risk, with rates reaching as high as 76% despite prompt surgical interventions. While polymicrobial infections were more likely to be encountered, few studies reported a monomicrobial predominance. Despite its relevance, there is a notable lack of data in the Eastern Mediterranean region. We aimed to investigate the microbiological profile among patients diagnosed with necrotizing fasciitis in a tertiary referral center in Lebanon, with a particular focus on Multidrug-resistant organisms (MDROs) and Antimicrobial Resistance (AMR), given their increasing prevalence and the challenges they pose in clinical management.

**Table 1:**
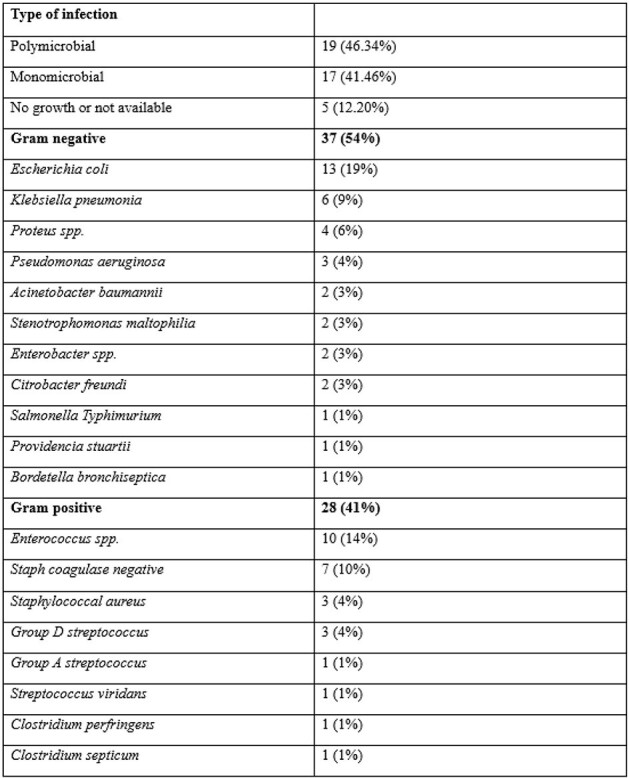
Distribution of Organisms

**Methods:**

A retrospective chart review of hospitalized cases between 2011 and 2021. The study population included adult patients diagnosed with NF during this period. Data included microbiological characteristics, with focus on Multidrug-resistant organisms (MDROs).

**Figure 1:**
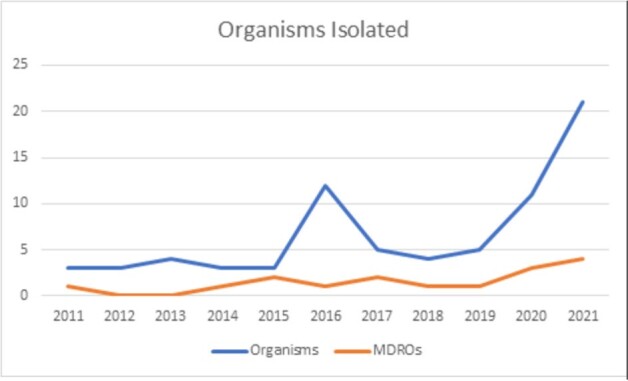
Yearly Trend of MDROs Among Isolated Organisms

**Results:**

41 cases of NF were identified showing a mortality rate of 37%. 46 % of cases had polymicrobial infections versus 42% monomicrobial. The predominant gram negative pathogen was *E.coli* in both types of infections followed by the gram positive pathogen, *Enterococcus species*. No difference in the type of infection was noticed in the survival group compared to a predominance of polymicrobial infection in the non-survival group. Fungi were recovered represented in 5.4% of cases and were part of a polymicrobial infection. Out of all the isolated organisms, 21.2% showed patterns of resistance (Extended Spectrum Beta Lactamase, Multidrug resistant, Vancomycin Resistant Enterococcus, and Carbapenem Resistant Enterobacteriaceae) with a yearly incremental rise.

**Conclusion:**

This 10-year study emphasizes the urgency of prompt surgical intervention and tailored antibiotic treatment for NF, particularly in the context of increasing AMR and the presence of MDROs, contributing crucial regional data on NF demographics, comorbidities, and microbiology

**Disclosures:**

**All Authors**: No reported disclosures

